# Promoter decoding of transcription factor dynamics

**DOI:** 10.1038/msb.2013.63

**Published:** 2013-11-05

**Authors:** Amie D Moody, Eric Batchelor

**Affiliations:** 1 Laboratory of Pathology, National Cancer Institute, National Institutes of Health, Bethesda, MD, USA

‘Steganography,' meaning ‘concealed writing,' is the art of hiding a secret message within another message. The original message appears to be simply a letter or photograph, yet a person with the requisite knowledge can decode it to find hidden information. As it turns out, cells may have developed a form of steganography. Recent studies have shown that a single transcription factor (TF) can exhibit different dynamics in response to different stimuli (Nelson et al, 2004; Tay et al, 2010; Batchelor et al, 2011; Hao and O'Shea, 2012) and that TF dynamics can alter target gene activation (Tay et al, 2010; Purvis et al, 2012). Hansen and O'Shea (2013) now provide insight into how individual promoters can decode specific TF dynamic patterns to effect distinct gene expression responses.

To understand this process, the authors studied Msn2, a yeast transcription factor that responds to different stresses with distinct dynamic expression patterns: short-duration repeated pulses, a single pulse with a dose-dependent duration, or a single pulse with a dose-dependent amplitude (Hao and O'Shea, 2012). Using a small molecule to control nuclear translocation of Msn2, Hansen and O'Shea (2013) challenged cells with a panel of 30 activation profiles simulating the natural Msn2 dynamics, covering a range of duration, amplitude, and number of pulses. To measure the effects of Msn2 dynamics on target gene expression, they generated diploid yeast strains in which genes encoding YFP or CFP replaced the ORFs on homologous chromosomes for seven strongly activated Msn2 target genes. Measuring fluorescence levels from the reporter strains treated with the Msn2 activation profiles demonstrated that promoters responded differently to either sustained Msn2 nuclear localization or pulses (Figure 1). Applying a three-state promoter model to the data, the authors identified two promoter classes: High amplitude threshold, Slow promoters (HS); and Low amplitude threshold, Fast promoters (LF). Of the seven promoters analyzed, three were classified as HS promoters, three as LF promoters, and one promoter was a hybrid that exhibited characteristics of both classes. The authors speculated that four classes of promoters might generally exist: those with either high or low amplitude thresholds (H or L) and either slow or fast (S or F) activation.

Numerous studies have shown that noise can play an important role in gene expression and cellular decisions, especially in unicellular organisms (Eldar and Elowitz, 2010; Munsky et al, 2012). To determine how noise impacts expression from the different promoter classes regulated by Msn2, Hansen and O'Shea (2013) used a dual-fluorescent protein system to measure both intrinsic and extrinsic noise for each of the seven promoters (Elowitz et al, 2002; Raser and O'Shea, 2004). Extrinsic noise reflects the variability arising from the shared environment, such as ribosome availability, whereas intrinsic noise results from the variability arising from sources unique to a particular promoter, such as stochastic binding events at specific binding sites. Analysis of noise in the Msn2 system revealed that slow promoters had higher total and intrinsic noise than fast promoters, and that oscillatory Msn2 activity was noisier than sustained Msn2 activation for both classes of promoters. Additionally, the probability of a promoter being activated was dependent on the intrinsic noise of the promoter. To explain this, the authors performed stochastic simulations to illustrate that for all pulses greater than 10 min, fast promoters are predicted to be activated in every cell. In contrast, even for pulses of a 50-min duration, the *in silico* analysis showed that slow promoters would be activated in only approximately half the cells. Further analysis revealed a strong correlation between the timescale of promoter activation and noise. A brief, low amplitude signal led to weak, but uniform, expression from LF promoters. A longer, higher amplitude signal generated a stronger, uniform expression. In contrast, in the absence of sufficient signal there was no expression from an HS promoter, and even after sufficient signal there was only heterogeneous expression from the promoter (Figure 1).

What determines whether a promoter is ‘fast' or ‘slow?' The authors hypothesized that the distinction is due to the speed of chromatin reorganization at the promoter. Examination of nucleosome structure on three of the seven promoters (one slow and two fast) showed that, indeed, chromatin remodeling occurred more quickly at fast promoters. Furthermore, knockouts of two different chromatin remodeling complexes (SWI/SNF and SAGA) led to a slower promoter activation and an increase in both intrinsic and total noise for a fast promoter.

Hansen and O'Shea (2013) provide intriguing evidence that a combination of amplitude threshold and promoter activation rate controls how TF dynamics is decoded at individual promoters, which can be grouped into four possible broad classes. The authors propose a link between promoter class and gene function for two of the promoters. For example, the HS promoter of *SIP18*, the product of which provides protection from reactive oxygen species, would only be activated during the long periods of Msn2 nuclear localization caused by oxidative stress. The LF promoter driving *HXK1*, a protein important for growth on non-fermentable carbon sources, is conversely primed for the quick response to glucose starvation; however, the link to function is not as clear for the other promoters tested. The tools and models described in this study provide a conceptual framework for understanding how promoters decode TF dynamics. Yet, exploring how these different promoter classes are used for different cellular functions and outcomes is a key unanswered question. Compiling the wealth of data available about TF binding affinities and dynamics, promoter architecture, and noise contributions into more realistic models may provide the next level of insight into how cells ‘decide' at what time and to what level gene expression is regulated.

## Figures and Tables

**Figure 1:**
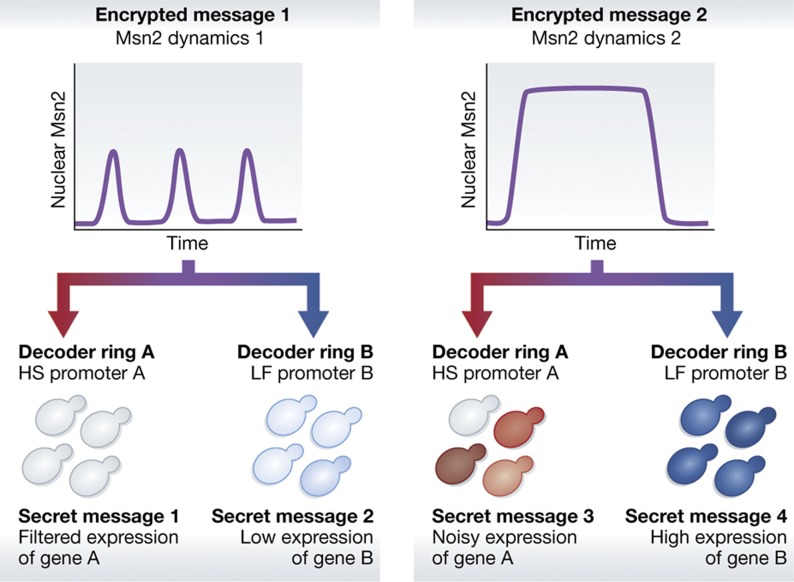
Msn2 shows complex dynamics that depend on the activating signal. The dynamics can be decoded differently at different promoters, leading to distinct patterns of gene expression and noise characteristics. Adapted from Hansen and O'Shea (2013).
